# Progesterone signaling in the regulation of luteal steroidogenesis

**DOI:** 10.1093/molehr/gaad022

**Published:** 2023-06-08

**Authors:** Kayhan Yakin, Francesko Hela, Ozgur Oktem

**Affiliations:** Graduate School of Health Sciences, Koç University, Istanbul, Turkey; School of Medicine, Department of Obstetrics and Gynecology, Koç University, Istanbul, Turkey; Graduate School of Health Sciences, Koç University, Istanbul, Turkey; Harvard Medical School, Islet Cell Biology and Regenerative Medicine, Joslin Diabetes Center, Boston, MA, USA; Graduate School of Health Sciences, Koç University, Istanbul, Turkey; School of Medicine, Department of Obstetrics and Gynecology, Koç University, Istanbul, Turkey

**Keywords:** progesterone, corpus luteum, pregnancy, luteotrophin, cholesterol, steroidogenesis, granulosa cell, apoptosis, angiogenesis

## Abstract

The corpus luteum is the major source of progesterone, the essential hormone for female reproductive function. While progesterone activity has been the subject of extensive research for decades, characterization of non-canonical progesterone receptor/signaling pathways provided a new perspective for understanding the complex signal transduction mechanisms exploited by the progesterone hormone. Deciphering these mechanisms has significant implications in the management of luteal phase disorders and early pregnancy complications. The purpose of this review is to highlight the complex mechanisms through which progesterone-induced signaling mediates luteal granulosa cell activity in the corpus luteum. Here, we review the literature and discuss the up-to-date evidence on how paracrine and autocrine effects of progesterone regulate luteal steroidogenic activity. We also review the limitations of the published data and highlight future research priorities.

## Introduction

This review article summarizes the current state of knowledge regarding the different facets of progesterone signaling in corpus luteum activity, covering its hormonal, paracrine, and autocrine actions. The notion that progesterone modulates its own secretion dates back to observations in the 1980s (Rothchild, 1981). Since then, progesterone activity in different tissues has become a subject of extensive research. Elegant studies have unraveled many of the complex signal transduction mechanisms exploited by the progesterone hormone. Collectively, these data indicate that the biological functions of progesterone are more versatile than previously envisioned. Both *in vivo* and *in vitro* studies in luteal cells as well as in different tissues are strongly supportive of the regulatory effect of progesterone.

Progesterone hormone is essential for female reproductive function. Deciphering its mechanism of action in target tissues can provide us with invaluable information to understand the pathophysiology of menstrual disorders, implantation failure, and early pregnancy complications.

## Methods

**Figure 1.**
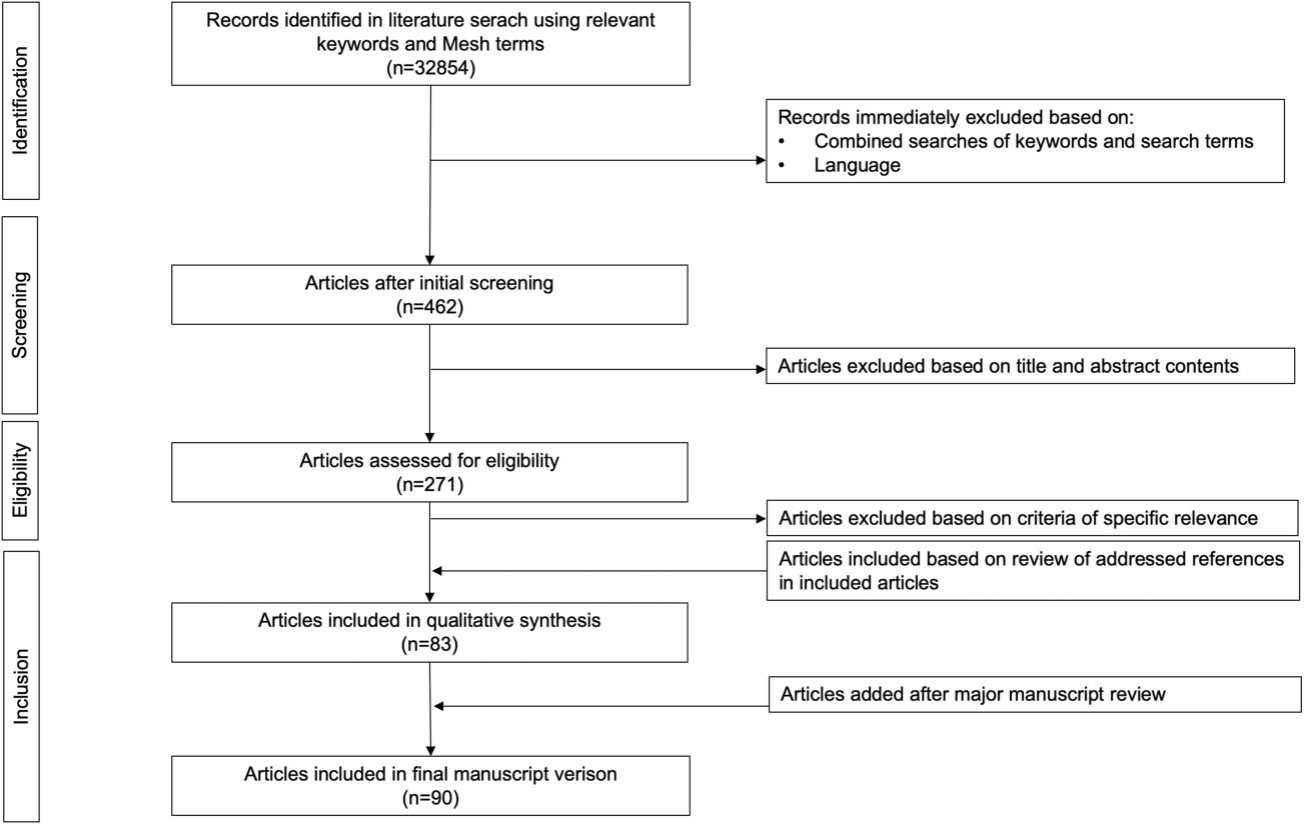
Schematic representation of literature search strategy and selection of relevant publications.

Our goal was to review the published data on progesterone activity in steroidogenic tissues. A comprehensive search for peer-reviewed articles in English was implemented based on articles available in Scopus, Web of Science, and PubMed databases, with no limits placed on time. The main terms ‘progesterone’, ‘progestin’, ‘granulosa cell’, ‘luteal cell’, ‘ovary’, ‘luteotrophin’, ‘autocrine’, and ‘paracrine’ were searched using Boolean operators ‘AND’, ‘OR’, and ‘NOT’ in various combinations. Experimental studies on human and animal tissues/cells, reviews, editorials, and commentaries were included. Duplicates or unrelated articles were excluded. Additional studies addressed by primary references and results from our own studies were included where relevant. The search strategy and selection steps are described in Fig. 1.

## Progesterone-induced signaling

Progesterone is synthesized from the parent molecule cholesterol through a series of complex chemical reactions catalyzed by steroid cytochrome P450 and hydroxysteroid dehydrogenases (Miller and Auchus, 2011). The source of cholesterol for steroidogenesis in ovarian luteal cells depends on circulating plasma lipoproteins, *de novo* synthesis, and utilization of intracellular cholesterol ester stores. Steroidogenic acute regulatory protein (StAR) facilitates the transport of cholesterol from the outer to the inner mitochondrial membrane, serving as the rate-limiting step in progesterone synthesis. P450 cholesterol side-chain cleavage enzyme (CYP11A) catalyzes the conversion of cholesterol to pregnenolone, which passes into the smooth endoplasmic reticulum where it is converted to progesterone by 3β-hydroxysteroid isomerase (3β-HSD). Progesterone then diffuses out of the luteal cell to be transported to the target tissues.

**Figure 2.**
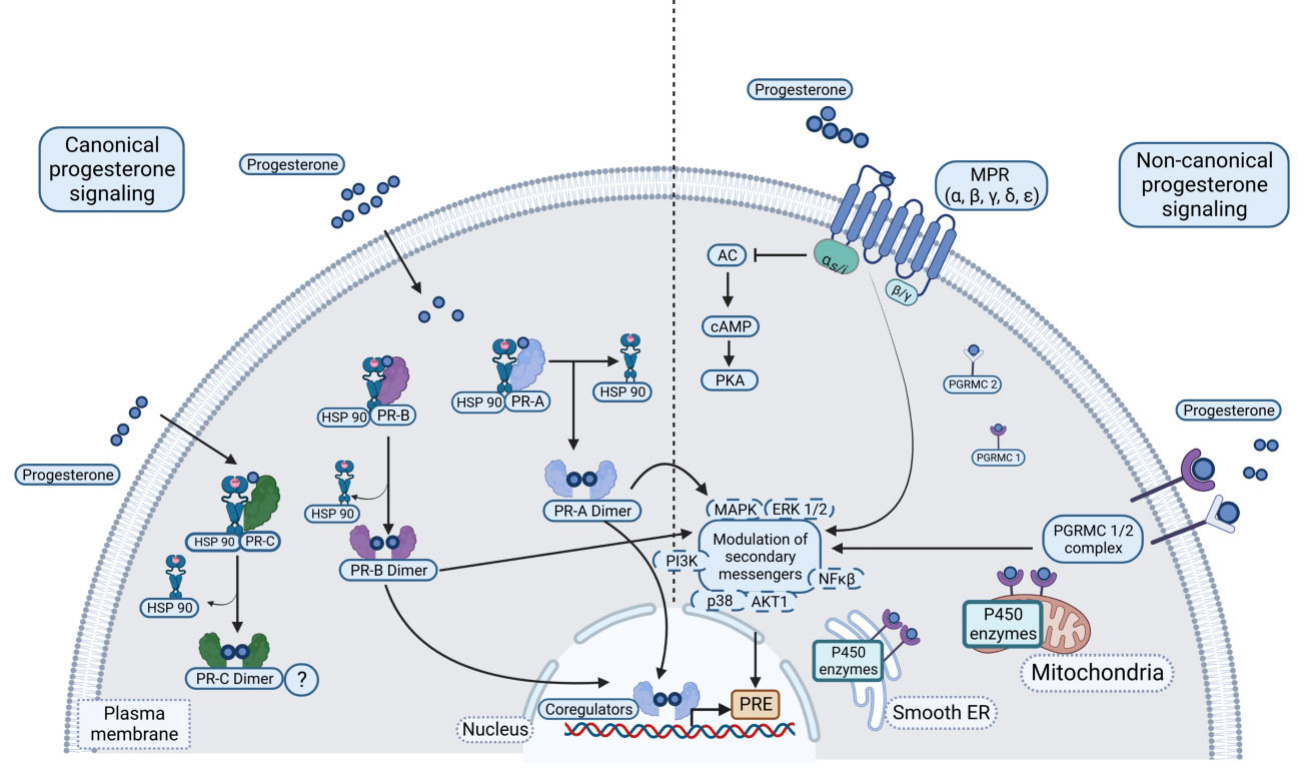
**Schematic summary of progesterone-induced receptor-signal transduction systems.** Canonical (or classical) progesterone receptors include progesterone receptor A (PR-A), B (PR-B), and C (PR-C). Non-canonical (nonclassical) progesterone signaling functions through both progestin and adiponectin Q receptors (MPRα, MPRβ, MPRγ, MPRð, and MPRɛ) and/or membrane-associated progesterone receptors such as the progesterone receptor membrane component 1 (PGRMC1) and 2 (PGRMC2). While ligand binding and downstream signal transduction cascades of canonical and non-canonical receptors differ in a tissue- and context-dependent manner, they seem to interact through secondary messengers (image created with Biorender.com). 20α-HSD: 20α-hydroxysteroid dehydrogenase; Bcl-2: B-cell lymphoma protein 2; Bad: Bcl-2-associated death promoter; INSIG1: insulin-induced gene 1; LCN-2: Lipocalin 2; LDL: low-density lipoprotein; MPR: membrane progesterone receptor; P450SCC: P450 cholesterol side-chain cleavage enzyme; PGRMC: progesterone receptor membrane component; PR: progesterone receptor; SCAP: sterol regulatory element-binding protein cleavage-activating protein; SRE: sterol regulatory element; SREBP: sterol regulatory element-binding protein; StAR: steroidogenic acute regulatory protein; VEGF: vascular endothelial growth factor.

Progesterone-induced gene transcription is mediated through different receptor/signal transduction pathways including classical (nuclear or canonical) receptors and nonclassical (membrane and membrane-associated or non-canonical) receptors (Fig. 2). Progesterone shows tissue-specific, context-specific, and menstrual phase-dependent activity. This context-dependent activity is achieved through the expression pattern and relative concentration of different types of progesterone receptors (PRs), interactions with co-regulatory factors (co-activators and co-inhibitors), cooperating with other transcriptional factors, and chromatin-dependent access to target genes. Moreover, while ligand binding and post-receptor signal transduction cascades vary and are complex, they seem to interact at several points but also have independent activities that lead to cell- and context-specific responses to progesterone (Peluso, 2006; Sueldo *et al.*, 2015).

### Nuclear (classical) PRs

The PRs belong to the nuclear/steroid hormone receptor family of ligand-dependent transcription factors. Following engagement with its ligand, a PR regulates the activity of a multitude of downstream effectors and the expression of target genes. Signaling through PRs induces a steadily growing and sustained response (Medina-Laver *et al.*, 2021).

Three major receptor isoforms, namely PR-A, PR-B, and PR-C, are encoded by the same gene but they are transcribed from different promoters (Misrahi *et al.*, 1993; Grimm *et al.*, 2016). An additional B upstream segment in the amino-terminal binding domain in PR-B includes an extra transactivation function domain, endowing PR-B a greater transcriptional activity than PR-A. The common N-terminal domain possesses a proline-rich sequence including a binding site for the Src homology 3 (SH3) domain of the Src tyrosine kinase. The third form, PR-C (45–50 kDa), which has been shown to be expressed in the pregnant uterus, can bind to the ligand but, owing to the lack of a DNA-binding domain, cannot bind to the progesterone response elements (PREs) of the target genes (Wei *et al.*, 1996).

The classical nuclear PRs are localized in the cytoplasm and the nucleus. In the absence of ligand, PR resides in complexes with chaperone molecules including heat shock proteins (HSP70 and HSP90). Ligand binding triggers a conformational change in the PR that leads to its dissociation from the HSP complex. Dimerized PR is rapidly transported to the nucleus and binds the chromatin-embedded genome where it acts as a transcription factor to regulate PREs of the responsive genes. Recruitment of several co-activators or co-inhibitors, including activator protein 1 (AP1), specificity protein1 (Sp1), and signal transducer and activator of transcription proteins (STATs), regulate the subsequent progesterone-induced target gene expression (Owen *et al.*, 1998). Moreover, progesterone modulates gene promoters that lack complete canonical PRE-binding sequences in association with other DNA-bound transcription factors (Faivre *et al.*, 2008) and cross-talk with the estrogen receptors (ER) (Migliaccio *et al.*, 1998). PR–chromatin interactions demonstrate remarkable tissue-specificity in terms of binding sites and interactions with other transcription factors (Dinh *et al.*, 2019).

In conjunction with their transcriptional activity, PRs also utilize the growth factor receptor signaling pathways. Ligand binding facilitates the association between proline-rich regions of the N-terminal domain and Src tyrosine kinases to activate the Src/Ras/Raf/Mek/Erk-1 cascade (Migliaccio *et al.*, 1998; Garg *et al.*, 2017). PR activity is controlled through phosphorylation by several kinases such as the cyclin-dependent kinase 1/2 (CDK1/2), mitogen-activated protein kinase (MAPK), protein kinase A (PKA), DNA-dependent protein kinase (DNA-PK), and casein kinase II (CK2) (Grimm *et al.*, 2016).

### Progestin and adiponectin Q receptors

Membrane progesterone receptors (MPRs) are seven-transmembrane G protein-coupled receptors with their N-termini facing toward the extracellular surface (Zhu *et al.*, 2003). They belong to the family of progestin and adiponectin Q receptors (PAQR). The expression of five isoforms (MPRα, β, γ, ð, and ɛ or PAQR7, PAQR8, PAQR5, PAQR6, and PAQR9) is cell-specific and hormone-dependent (Medina-Laver *et al.*, 2021).

MPRs bind progesterone with high affinity (Kd of 20–40 nM) but do not bind to synthetic progesterone agonists or antagonists such as mifepristone. Ligand activation increases tyrosine kinase and MAPK activities and decreases adenylate cyclase activity. Moreover, MPRs participate in other signal transduction pathways including extracellular signal-regulated kinases 1/2 (Erk1/2) or p38 MAPKs. (Charles *et al.*, 2010).

### Membrane-associated PRs

The membrane-associated PR family comprises four homologous proteins: progesterone receptor membrane component 1 (PGRMC1), progesterone receptor membrane component 2 (PGRMC2), neudesin, and neuferricin (Kimura *et al.* 2012). These proteins possess a similar non-covalent heme/steroid-binding domain, which is related to the cytochrome b5, a crucial component of microsomal cytochrome P450 monooxygenase systems.

The PGRMC1 protein comprises a single transmembrane domain, a short extracellular domain, and a cytoplasmic tail (Gerdes *et al.*1998; Cahill, 2007; Peluso, 2007). It binds and interacts with plasminogen activator inhibitor RNA binding protein-1 (PAIRBP-1) (Peluso *et al.*, 2005, 2006). PGRMC2 harbors a structurally similar cytochrome b5 domain to PGRMC1 but a different N-terminal transmembrane domain. Both PGRMC1 and PGRMC2 possess several Src homology target sequences for molecular interactions with other intracellular proteins that contain SH2 and SH3 domains (Cahill, 2007; Cahill *et al.*, 2016).

The two other members of this receptor family, neudesin and neuferricin, are involved in the regulation of neural activity but no functions related with progesterone have been reported to date (Ryu *et al.*, 2017).

Unlike nuclear PRs, which mainly modify transcriptional activity to build a gradually emerging cellular response, non-canonical signaling induces an array of secondary messengers and signal transduction pathways that lead to an immediate response upon ligand binding (Medina-Laver *et al.*, 2021).

Unlike PR-A and PR-A, PGRMC1 and MPRs do not bind to RU 486 (Peluso, 2006). Upon ligand binding, PGRMC1 interacts with PGRMC2 and functions as a membrane receptor complex (Peluso, 2006). As PGRMC1 interacts with both steroidogenic and drug-metabolizing cytochrome P450 enzymes, it is localized in various locations that correspond to its proposed function including the mitochondria, smooth endoplasmic reticulum, plasma membrane, cytoplasm, and nucleus (Piel *et al.*, 2016). PGRMC2 mainly localizes to the cytoplasm (Peluso *et al.*, 2019). Recent studies on breast tissue reveal a crosstalk between PGRMC1 and ERα. Silencing PGRMC1 attenuated ERα expression in breast cancer cells (Pedroza *et al.*, 2021). Progestins, including norethisterone and dydrogesterone, facilitated the interaction between PGRMC1 and prohibitins which, in turn, decreased binding of prohibitins to ERα and increased ERα activation (Bai *et al.*, 2021).

The characteristics of receptors involved in progesterone-induced signaling in ovarian cells are summarized in Table 1. The expression profile of these receptors varies among species. While PRA and PRB expression is not detectable in rat corpus luteum, both isoforms appear in the granulosa cells of preovulatory follicles in humans and Rhesus monkey and continue throughout the luteal phase of the cycle, in correlation with the serum progesterone level. In contrast, in the Rhesus monkey, PRA protein levels gradually decline during the luteal phase while PRB levels remain relatively constant (Misao *et al.*, 1998; Ottander *et al.*, 2000; Peluso *et al.*, 2006).

**Table 1:** The characteristics of receptors involved in progesterone-induced signaling in ovarian cells and their expression profile through the menstrual cycle.

Receptor family	Isoform	Gene locus (chromosome)	Molecular weight (kDa)	Progesterone binding constant (nM)	Expression in granulosa cells		
					Follicular	Periovulatory follicular	Luteal
**Nuclear progesterone receptors**	A	11	94	1–5	−	−	+*
	B	11	114	1–5			

**Progestin and adiponectin Q receptors**	MPRα	1	40	20–40	ND	+**	+
	MPRβ	6	40	20–40			
	MPRγ	15	40	20–40			

**Membrane -associated progesterone receptors**	PGRMC1	X	22	35	+	+	+
	PGRMC2	4	24	–			

MPR: membrane progesterone receptor, PGRMC: progesterone receptor membrane component,.

* Species-specific, not detected in rats.

** Detected in Medaka fish: all other data are from human. ND: not determined.

**Table 2:** Summary of the key experimental studies related to proposed mechanisms involved in the luteotrophic activity of progesterone.

	Reference	Species	Cell/tissue	Study outcome
**Progesterone inhibits apoptosis and supports survival of granulosa/luteal cells**	Makrigiannakis *et al.* (2000)	Human	Granulosa cells	Progesterone reduces and RU 486 increases the rate of apoptosis. RU 486 inhibits the protective effect of progesterone on survival.
	Chaffin *et al.* (2001)	Macaque	Granulosa cells	Suppression of progesterone levels through inhibition of 3βHSD by trilostane increases apoptosis which can be reversed by progesterone supplementation
	Mussche and D’Herde (2001)	Quail	Granulosa cells	Blocking progesterone synthesis by aminoglutethimide or progesterone receptors by RU 486 causes an increase in the rate of apoptosis
	Shao *et al.* (2003)	Mouse	Granulosa cells	RU 486 treatment augments caspase-3 activation
	Rung *et al.* (2005)	Rat and human	Granulosa cells	Inhibition of progesterone signaling and/or synthesis increases apoptosis, which is reversed by progesterone administration
	Peluso *et al.* (2005)	Rat	Luteal/granulosa cells	PAIRBP1 mediates progesterone’s antiapoptotic activity and this activity might involve interaction with PGRMC1
	Liszewska *et al.* (2005)	Cow	Luteal cells	Progesterone treatment increases the expression of the anti-apoptotic factor Bcl-2
	Peluso *et al.* (2006)	Rat	Luteal/granulosa cells	PGRMC1 interacts with PAIRBP1 to form a complex that is required to exert its anti-apoptotic action
	Engmann *et al.* (2006)	Human	Luteal/granulosa cells	Progesterone suppresses the rate of apoptosis
	Peluso *et al.* (2007)	Rat	Granulosa cells	Progesterone promotes survival through regulation of PKG and Mek/Erk activities, as induction of PKG activity by 8-br-cGMP supports and inhibition of PKG by delta-tocotrienol suppresses progesterone’s anti-apoptotic effects
	Peluso *et al.* (2008)	Rat	Granulosa cells	siRNA-induced knockdown of PGRMC1 prevents progesterone from inhibiting apoptosis
	Peluso *et al.* (2009)	Human	Granulosa cells	Progesterone activates a PGRMC1-dependent mechanism to promote survival
	Peluso *et al.* (2010)	Rat	Granulosa cells	Progesterone supports cell survival by increasing the Bcl21d to Bad ratio
	Peluso *et al.* (2012)	Human	Granulosa cells	Progesterone downregulates caspase-3 expression
	Quirk *et al.* (2013)	Cow	Luteal cells	Progesterone treatment decreases Fas-ligand induced apoptosis
	Pietrowski *et al.* (2014)	Human	Luteal cells	Progesterone treatment downregulates Fas expression
	Griffin *et al.* (2014)	Rat	Granulosa cells	Progesterone slows down the rate of apoptosis through PGRMC2
	Peluso *et al.* (2014)	Rat	Granulosa cells	siRNA-induced depletion of Pgrmc1 or antibody-induced disruption of PGRMC1/2 complex increases the rate of apoptosis

**Progesterone augments its own synthesis**	Uchida *et al.* (1970)	Rat	Granulosa cells	Exogenous progesterone augmented progesterone secretion
	Fortune and Vincent (1983)	Mice	Granulosa cells	Progesterone regulates *in vitro* steroidogenic activity
	Fanjul *et al.* (1983)	Rat	Granulosa cells	Synthetic progestin R5020 increases gonadotropin-induced progesterone synthesis
	Ruiz de Galarreta *et al.* (1985)	Rat	Granulosa cells	Natural and synthetic progestins augment pregnenolone and progesterone production via increasing 3β-HSD activity
	Hibbert *et al.* (1996)	Monkey	Luteal cells	Inhibition of 3β-HSD induces structural changes similar to luteal regression, which is reversed by addition of the synthetic progesterone R5020
	VandeVoort *et al.* (2000)	Human	Luteal/granulosa cells	Progesterone antagonists RU 486 and HRP 2000 suppress ovarian steroid synthesis
	Ottander *et al.* (2000)	Human	Luteal/granulosa cells	Progesterone exerts a stimulatory action on steroid synthesis through its receptors
	Yang *et al.* (2002)	Human	Kidney cells	PGRMC1/SCAP/INSIG1 complex activates transcriptional activity of SREBP and induces the expression of steroidogenic genes, including StAR
	Suchanek *et al.* (2005)	Monkey	Kidney cells	PGRMC1 interacts with SCAP and INSIG1
	Rekawiecki *et al.* (2005)	Cow	Luteal/granulosa cells	Progesterone increases mRNA expression of StAR, 3β-HSD, and CYP19A1
	Peluso *et al.* (2009)	Human	Granulosa cells	Synthetic progesterone R5020 stimulates progesterone secretion
	Yao *et al.* (2020)	Sheep	Luteal/granulosa cells	Progesterone increases mRNA expression of StAR, 3β-HSD, and CYP19A1

**Progesterone modulates* de novo* cholesterol synthesis **	Rung *et al.* (2005)	Rat and human	Granulosa cells	Progesterone receptor antagonists Org 31710 and RU 486 suppress *de novo* cholesterol synthesis which is reversed by addition of progesterone
	Hughes *et al.* (2007)	Human	Kidney cells	PGRMC1 binds to INSIG1 and modulates CYP51A1 family members that control cholesterol synthesis
**Progesterone promotes luteal vascularization **	Shimizu and Miyamoto (2007)	Cow	Granulosa and theca cells	Progesterone augments the expression of VegfA120
	Peluso *et al.* (2018)	Mice	Luteal/granulosa cells	Vascularization of corpus luteum is attenuated in PGRMC1/2 deleted mice
	Peluso *et al.* (2018)	Mice	Luteal/granulosa cells	Inhibition of PGRMC1/2 activity is associated with reduction in the number of vascular endothelial cells
	Nichols *et al.* (2019)	Cattle	Granulosa and theca cells	Progesterone augments the expression of Vegf-A
	Narimani *et al.* (2020)	Mice	Granulosa and theca cells	Progesterone augments the expression of Vegf and angiogenic parameters including vascular density and area

**Progesterone regulates lipoprotein uptake **	Furuhata *et al.* (2020)	Mice	Adipocytes	PGRMC1 interacts with VLDL-R and LDL-R or GLUT4 to facilitate the uptake of lipoproteins
	Oh *et al.* (2013)	Mice	Not specified	Progesterone upregulates low-density lipoprotein receptor-related protein 2
	Riad *et al.* (2018)	Human	HeLa cells	PGRMC1 forms a complex with TMEM97 and LDL-R to assist the uptake of LDL and apolipoprotein E

**Progesterone suppresses its own catabolism**	Sugino *et al.* (1997)	Rodent	Luteal cells	Progesterone suppresses its own catabolism by downregulating the expression of 20α-HSD
	Fortune and Vincent (1983)	Rodent	Granulosa cells	Progesterone suppresses the conversion of progesterone to estrogen by inhibiting aromatase activity
	Beranic *et al.* (2011)	Human	Endometrium	Progesterone inhibits aldo-ketoreductases 1C1 and 1C3 which convert progesterone to the less potent metabolite 20α-hydroxyprogesterone

**Progesterone modulates the expression of progesterone receptors **	Misao *et al.* (1998)	Human	Luteal cells	High concentration of progesterone induces PR-A expression, which suppresses PR-B expression and subsequently decreases progesterone activity
	Chaffin *et al.* (1999)	Macaque	Granulosa cells	Steroid depletion reduces progesterone receptor expression in periovulatory granulosa cells which is reversed by progesterone replacement
	Guerra-Araiza *et al.* (2002)	Rat	Brain tissues	Progesterone shows a gender-dependent and tissue-specific effect on the expression of PR isoforms
	Gordon *et al.* (2015)	Rat	Brain tissues	Progesterone shows a time- and dose-dependent effect on the expression of PR isoforms
	Diep *et al.* (2016)	Human	Breast, endometrial, and ovarian cancer cell lines	High doses of synthetic progestin R5020 upregulate PR expression which is reversed by the addition of an antiprogestin RU 486

3β-HSD: 3β-hydroxysteroid isomerase; 8-br-cGMP: 8-bromo-cyclic GMP; 20α-HSD: 20α-hydroxysteroid dehydrogenase; CYP19A1: cytochrome P450 family 19 subfamily A member 1; CYP51A1: cytochrome P450 family 51 subfamily A member 1; GLUT4: glucose transporter type 4; INSIG1: insulin-induced gene 1; LDL: low-density lipoprotein receptor; PAIRBP-1: plasminogen activator inhibitor RNA binding protein -1; PGRMC1: progesterone receptor membrane component 1; PGRMC2: progesterone receptor membrane component 2; PKG: cyclic GMP-dependent kinase; PR-A: progesterone receptor A, PR-B: progesterone receptor-B; SCAP: sterol regulatory element binding protein cleavage- activating protein; SERBP-1: serpine 1 mRNA binding protein; SREBP: sterol regulatory element binding protein; StAR: steroidogenic acute regulatory protein; TMEM97: sigma-2 receptor/transmembrane protein 97; VEGF-A: vascular endothelial growth factor-A; VLDL: very low-density lipoprotein receptor.

PGRMC2 and PAIRBP1 are expressed in granulosa cells throughout the menstrual cycle in humans (Sueldo *et al.*, 2015) and their expression level was reported to be increased in the luteal phase in a rat model (Peluso *et al.*, 2006; Griffin *et al.*, 2014). While MPRα, MPRβ, and MPRγ expression was shown in rat corpus luteum and granulosa cells of Medaka fish preovulatory follicles (Hagiwara *et al.*, 2016), human data are lacking (Cai and Stocco, 2005).

Human luteal granulosa cells express PR-A, PR-B, PGRMC1, PGRMC2, and MPRα, β, γ, and ð at different levels (Peluso *et al.*, 2009; Pietrowski *et al.*, 2014; Sueldo *et al.*, 2015). While significant patient variation has been detected in their relative expression, PGRMC1 is the most abundantly expressed receptor, followed by MPRα, PGRMC2, and PR, when compared to actin expression (Sueldo *et al.* 2015).

## Progesterone-modulated mechanisms in luteal granulosa cell activity

**Figure 3.**
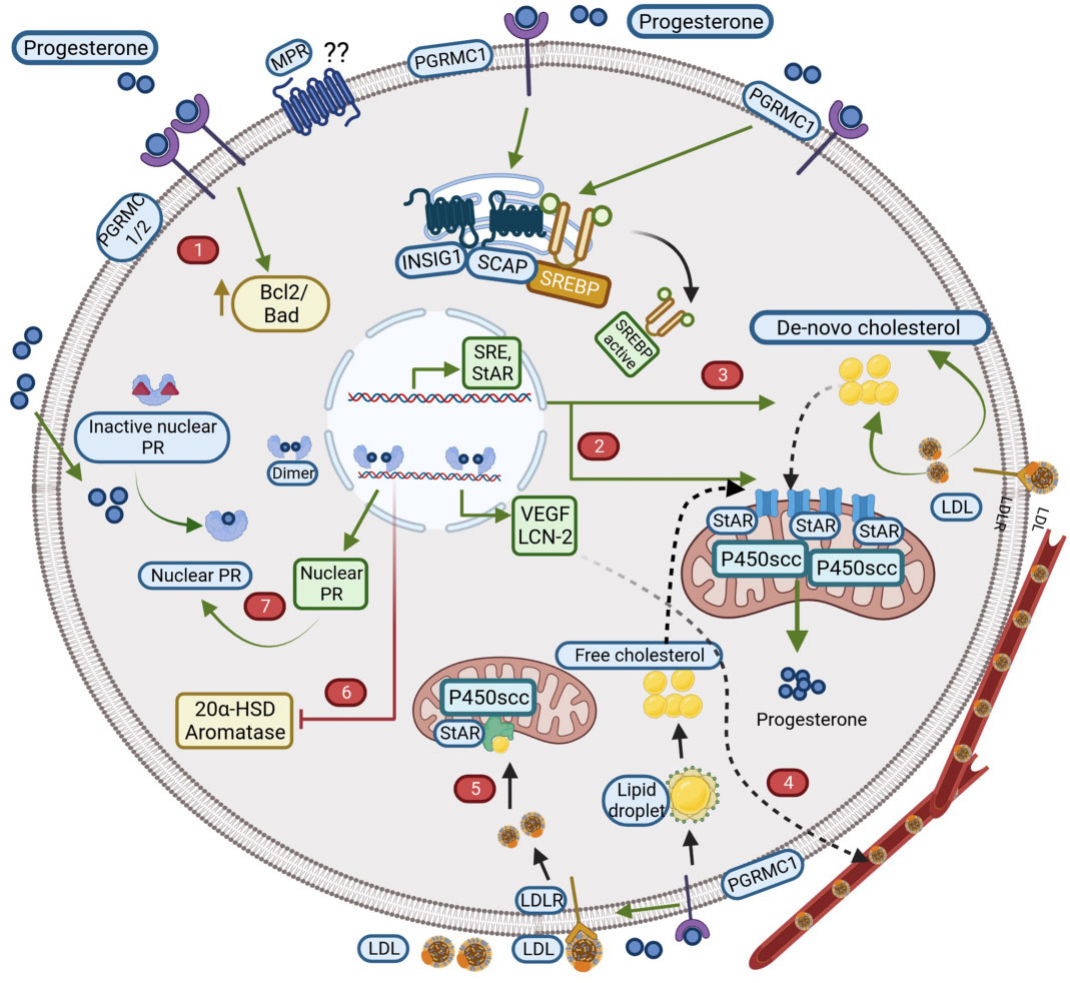
**Progesterone modulates luteal activity on granulosa cells through different mechanisms.** The different mechanisms are: (1) suppression of apoptosis and supporting the survival of granulosa cells, (2) supporting its own synthesis through induction of steroidogenic enzymes, (3) regulating *de novo* cholesterol synthesis through interaction of PGRMC1 with INSIG1 and SCAP, (4*) supporting luteal vascularization through angiogenic modulators such as VEGF and lipocalin, (5*) promoting lipoprotein uptake, (6) suppressing its own catabolism through inhibition of 20α-hydroxysteroid dehydrogenase, and (7) increasing its own receptor expression. * denotes functions yet to be demonstrated in ovarian granulosa cells (image created with Biorender.com). AC: adenylyl cyclase; AKT: Ak strain transforming protein; cAMP: cyclic adenosine monophosphate; ER: endoplasmic reticulum; ERK: extracellular signal-regulated kinases; HSP: heat shock protein; INSIG1: insulin-induced gene 1; MAPK: mitogen-activated protein kinase; MPR: membrane progesterone receptor; NFκB: nuclear factor kappa-light-chain-enhancer of activated B cell; PGRMC1: progesterone receptor membrane component 1; PI3K: phosphoinositide 3-kinase; PKA: protein kinase A; PR: progesterone receptor; PRE: progesterone response elements; SCAP: sterol regulatory element-binding protein cleavage-activating protein; VEGF: vascular endothelial growth factor

There is considerable evidence showing that progesterone supports luteal granulosa cell survival and regulates its own synthesis through different mechanisms (Fig. 3). Table 2 provides a summary of the key experimental studies related to the proposed mechanisms of progesterone’s luteotrophic activity.

### Progesterone suppresses apoptosis and supports the survival of granulosa cells

Several studies indicate that progesterone promotes luteal activity by supporting the survival of granulosa/luteal cells and by inhibiting their apoptosis. *In vitro* studies in animal and human periovulatory granulosa cells show that inhibition of progesterone signaling and/or synthesis increases apoptosis, which can be reversed by administration of progesterone (Makrigiannakis et al., 2000; Mussche and D’Herde, 2001; Rung *et al.*, 2005). Progesterone’s anti-apoptotic activity might occur through different mechanisms. For example, progesterone treatment decreases Fas ligand-induced apoptosis in cow corpus luteum (Quirk *et al.*, 2013) and in luteal granulosa cells recovered from infertile women (Pietrowski *et al.*, 2014). In a human granulosa cell line (hGL5 cells), progesterone suppresses the expression of the apoptosis activator caspase-3 (Peluso *et al.*, 2012) while in mouse granulosa cells, RU-486 treatment was associated with an increase in caspase-3 activation (Shao *et al.*, 2003). Progesterone treatment increased the expression of anti-apoptotic B-cell-lymphoma-2 protein (Bcl-2) in bovine luteal cells (Liszewska *et al.*, 2005) and favored cell survival in spontaneously immortalized granulosa cells (SIGC) by increasing the Bcl21d to Bcl-2 associated death promoter ratio (Peluso *et al.*, 2010). In freshly recovered human luteal granulosa cells and spontaneously immortalized rat granulosa cells, progesterone promotes survival through regulation of cyclic GMP-dependent kinase (PKG) and Mek/Erk activities, as induction of PKG activity by 8-br-cGMP supports, and inhibition of PKG by delta-tocotrienol suppresses, progesterone’s anti-apoptotic effects (Peluso *et al.*, 2006, 2007).

The mechanism of how progesterone attenuates apoptosis and promotes cell survival, even in cells that do not express classical PRs, is explained by the outstanding mechanistic analyses reported by Peluso’s team, who demonstrated that PGRMC1 is responsible for the pro-survival effects of progesterone in human and rat granulosa/luteal cells (Peluso *et al.*, 2005, 2008, 2009, 2012, 2014; Griffin *et al.*, 2014). While progesterone favored cell survival in SIGC (Peluso *et al.*, 2010), siRNA-induced depletion of Pgrmc1 or antibody-induced disruption of the PGRMC1/2 complex was associated with an inappropriate entry into the cell cycle, which resulted in an increased rate of apoptosis (Peluso *et al.*, 2014).

### Progesterone increases its own synthesis by StAR-induced steroidogenesis

Research on the characterization of progesterone’s role in the formation and maintenance of the corpus luteum dates back to the early 1970s. Administration of exogenous progesterone augmented progesterone secretion from rat ovaries (Uchida *et al.*, 1970). In addition, progesterone was shown to regulate *in vitro* steroidogenic activity of the granulosa cells of mice (Fortune and Vincent, 1983). The synthetic progestin R5020 increased the gonadotrophin-induced progesterone synthesis in cultured rat granulosa cells (Fanjul *et al.*, 1983). Likewise, in the same setting, both natural and synthetic progestins augmented pregnenolone and progesterone production via increasing 3β-HSD activity (Ruiz de Galarreta *et al.*, 1985). Inhibiting progesterone synthesis by the 3βHSD inhibitor trilostane induced structural changes similar to those seen in luteal regression in Rhesus monkeys, which was reversed by the addition of R5020 (Hibbert *et al.*, 1996). Based on these data in 1996, Rothchild proposed that intraovarian progesterone might function as ‘the universal luteotropin’, to promote its own secretion (Rothchild, 1996). Subsequent studies provided further molecular evidence to explain Rothchild’s proposal for the autocrine effects of progesterone to stimulate its own synthesis by the corpus luteum (VandeVoort *et al.*, 2000; Ottander *et al.*, 2000; Stouffer, 2003; Rung *et al.*, 2005; Peluso *et al.*, 2009). Progesterone increased the mRNA expression of StAR, 3β-HSD, and CYP19A1 in cow (Rekawiecki *et al.*, 2005) and sheep (Yao *et al.*, 2020) luteal/granulosa cells.

The identification of non-canonical progesterone signaling in granulosa cells helped to explain the molecular mechanism of progesterone enhancing its own synthesis in luteal cells, which are known to be devoid of classical PRs (Peluso and Pru, 2014). Elegant studies demonstrated that PGRMC1 is likely to be involved in progesterone-induced augmentation of steroid synthesis (Peluso and Pru, 2014). PGRMC1 interacts with two critical proteins that regulate steroidogenesis; sterol regulatory element binding protein cleavage-activating protein (SCAP) and insulin-induced gene 1 (INSIG1) (Suchanek *et al.*, 2005). This complex activates transcriptional activity of sterol regulatory element binding protein (SREBP) to induce the expression of various genes involved in steroidogenesis, including the key enzyme StAR (Christenson and Strauss, 2001; Yang *et al.*, 2002).

Interestingly, the capacity of luteal granulosa cells to secrete progesterone was not altered when any of the classical and nonclassical PRs were selectively depleted through siRNA treatment (Sueldo *et al.*, 2015). The most plausible explanation is that, despite sharing one or more common signaling pathways, each of these modulators regulates separate and independent transduction pathways to maintain progesterone secretion.

Another interesting topic that warrants further research is the association of PRs with autophagy modulators. In cancer cell lines, PGRMC1/2 was shown to bind microtubule-associated protein 1A/1B light chain 3B, a critical protein that is necessary for the formation of autophagosomes (Mir *et al.*, 2013). Autophagy is known to have an important role in the steroidogenic activity of steroid-producing cells (Tang *et al.*, 2019). Further research is necessary to dissect the role of PGRMC in the autophagic machinery of luteal granulosa cells.

### Progesterone signaling is associated with *de novo* cholesterol synthesis in granulosa cells

Several lines of evidence suggest that progesterone is involved in cholesterol sensing and synthesis mechanisms in the ovarian granulosa cells. For instance, the PR antagonists Org 31710 and RU 486 suppressed *de novo* cholesterol synthesis in rat and human granulosa cells (Rung *et al.*, 2005) and this could be reversed by the addition of progesterone. As the genes that regulate cholesterol synthesis do not contain functional PREs, these findings were proposed to be related to SREBP-2. The most convincing evidence regarding progesterone’s role in cholesterol metabolism is derived from a study that showed that PGRMC1 binds to INSIG1 and modulates CYP51A1 family members that control cholesterol synthesis (Hughes *et al.*, 2007). Based on the interaction of PGRMC1 with INSIG1 and SCAP, as well as circumstantial evidence from a non-peer-reviewed study, Cahill and Medlock suggested that PGRMC1 might be involved in cholesterol sensing and trafficking through different compartments of the cell by acting as a cholesterol-chaperone (Warnick, 2008; Cahill and Medlock, 2017). PGRMC1 appears to function as a versatile protein in cholesterol trafficking and steroid synthesis, and many of its functions remain to be studied in detail.

### Progesterone promotes luteal vascularization

In the midluteal phase of the menstrual cycle, the corpus luteum has the capacity to augment progesterone synthesis by several orders of magnitude (Taraborrelli, 2015), maintaining a progesterone output of around 40 mg per day (MacDonald *et al.*, 1991). This requires a significant increase in the availability of cholesterol for steroid synthesis. An increase in luteal vascularization would facilitate lipid uptake from the circulation and support the increased demand. Progesterone seems to support its own synthesis by inducing luteal vascularization (Curry and Nothnick, 1996; Pall *et al.*, 2000; Stouffer, 2003; Peluso *et al.*, 2018). Progesterone is known to increase the expression of the vascular endothelial growth factor (VEGF) gene in ovarian tissue in mice (Narimani *et al.*, 2020), granulosa and theca cells in ewes (Narimani *et al.*, 2020), cows (Shimizu and Miyamoto, 2007), and cattle (Nichols *et al.*, 2019). Inhibition of PGRMC1/2 activity is associated with a reduction in the number of vascular endothelial cells within the mouse corpus luteum (Peluso *et al.*, 2018), This effect seems to be independent of VEGF-A but rather related to another novel angiogenesis regulator, lipocalin 2, as its expression in corpus luteum is dependent on the presence of PGRMC1/2. In different target tissues other than the ovaries, progesterone exploits multiple signaling pathways to regulate neovascularization, including basic fibroblast growth factor, angiopoietin 1 and 2, hypoxia inducible factor α, and nitric oxide (Xia *et al.*, 2021). However, involvement of these factors in the augmented vascularization of the corpus luteum has yet to be determined.

### Progesterone regulates lipoprotein uptake

No less important than its role in amplified steroidogenic enzyme activity, progesterone may also increase the availability of lipoprotein-derived cholesterol substrate for steroid synthesis. In adipocytes PGRMC1 interacts with low-density lipoprotein receptors (VLDL-R and LDL-R) or glucose transport protein 4 (GLUT4) to facilitate the uptake of lipoproteins (Furuhata *et al.*, 2020). In murine uterus, high-density DNA microarray analysis indicated that progesterone upregulates LDL receptor-related protein 2 (*Lrp2*) (Oh *et al.*, 2013). CRISPR-induced gene knockout studies in HeLa cells revealed that PGRMC1 forms a complex with sigma-2 receptor/transmembrane protein 97 (TMEM97) and LDL-R to assist the uptake of LDL and apolipoprotein E (Riad *et al.*, 2018). The intriguing question of whether progesterone promotes lipoprotein uptake in luteal granulosa cells has not yet been studied.

### Progesterone suppresses its own catabolism

Progesterone seems to regulate its own catabolism through different mechanisms. The human aldo-ketoreductase (AKR) subfamily member 20α-hydroxysteroid dehydrogenase (20α-HSD) reduces progesterone to its inactive metabolites, 20α-hydroxyprogesterone, 20α-hydroxy-4-pregnen-3-one, and other weak progesterones (Penning *et al.*, 2004; Chu *et al.*, 2022). In rodent luteal cells, progesterone was shown to suppress its own catabolism by downregulating the expression of 20α-HSD enzyme (Sugino *et al.*, 1997). In rodent granulosa cells, progesterone suppresses the conversion of progesterone to estrogen by inhibiting aromatase activity (Fortune and Vincent, 1983). In human endometrium, progesterone and its derivatives (dydrogesterone, 20α-hydroxydydrogesterone, medroxyprogesterone acetate, desogestrel, norethindrone, and levonorgestrel) were shown to inhibit AKR1C1 and AKR1C3 (Beranic *et al.*, 2011).

### Progesterone modulates the expression of PRs

The current understanding is that progesterone downregulates the expression of PRs (Park and Mayo, 1991). However, Stouffer challenged this concept, at least for the granulosa cell, suggesting that recovery of PR expression following progesterone treatment in macaque granulosa cells after blockage with trilostane plus hCG shows that progesterone may upregulate PR expression, at least in early luteinization (Chaffin *et al.*, 1999; Stouffer, 2003). The idea is supported by another study, which showed that a high concentration of progesterone in luteal cells induced PR-A expression, which suppresses PR-B expression and subsequently decreased progesterone activity (Misao *et al.*, 1998). In the brain tissues of gonadectomized rats, progesterone showed a gender-dependent and tissue-specific (Guerra-Araiza *et al.*, 2002) as well as time- and dose-dependent (Gordon *et al.*, 2015) effect on the expression of PR isoforms. In PR^+^ERα^+^ breast, endometrial, and ovarian cancer cell lines, high doses of the progestin R5020 upregulated PR expression at the transcriptional and translational levels and the effect was reversed by the addition of the antagonist RU 486 (Diep *et al.*, 2016). These data indicate that the impact of progesterone on PR gene expression is more complex than has been reported. It is of note that the available data are concentrated on the expression of classical (nuclear) PRs. The effect of progesterone on the expression of membrane and membrane-associated receptors in the luteal granulosa cells warrants further research.

## Conclusions

A functional corpus luteum is essential for endometrial preparation, implantation, and the maintenance of early gestation. Despite extensive research, many questions regarding the intricate mechanism(s) that control luteinization, luteolysis, and the luteo-placental shift remain to be answered. Studies exploiting the advantages of progesterone antagonists and gene knockout/knockdown technologies and characterization of non-canonical receptor/signal transduction pathways have helped the research on progesterone gain momentum in the last two decades. Readers are referred to the excellent reviews on classical and nonclassical progesterone signal transduction, chromatin interactions, and regulation of transcription for further details (Peluso and Pru, 2014; Grimm *et al.*, 2016; Garg *et al.*, 2017; Cahill and Medlock, 2017; Ryu *et al.*, 2017; Dinh *et al.*, 2019; Medina-Laver *et al.*, 2021). Recent data provide further evidence and reiterate the long-held claim that the corpus luteum is not only the major site of progesterone synthesis but also a target tissue where progesterone regulates luteal granulosa cell activity through diverse mechanisms (Rothchild, 1996). Some of the mechanisms have been well-characterized in human corpus luteum. However, many have been studied in animal models or in tissues other than the ovaries. Caution should be exercised when extrapolating findings from *in vitro* studies, and species-specific, tissue/cell-specific, and menstrual phase-dependent differences in progesterone activity need to be taken into consideration. A significant majority of studies on human granulosa cells were based on luteal granulosa cells isolated from the follicular fluid obtained during oocyte retrieval in women who had undergone ART, unless otherwise specified. These mural luteinized granulosa cells of the early stage-corpus luteum may not truly represent pure natural cycles where the mid-cycle surge of endogenous LH triggers the ovulation. In ART, women are subjected to gonadotrophin treatment for ovarian stimulation and hCG or a GnRH analog for induction of final oocyte maturation. Both ovarian stimulation and the mode of triggering of oocyte maturation are associated with significant alterations in the viability and steroidogenic capacity of luteal granulosa cells (Bildik *et al.*, 2019). Moreover, cholesterol uptake/trafficking and progesterone synthesis by luteal granulosa cells are also related to maternal age and ovarian response to gonadotrophin stimulation (Bildik *et al.*, 2022). These findings should be considered when interpreting the findings of such studies.

**Figure 4.**
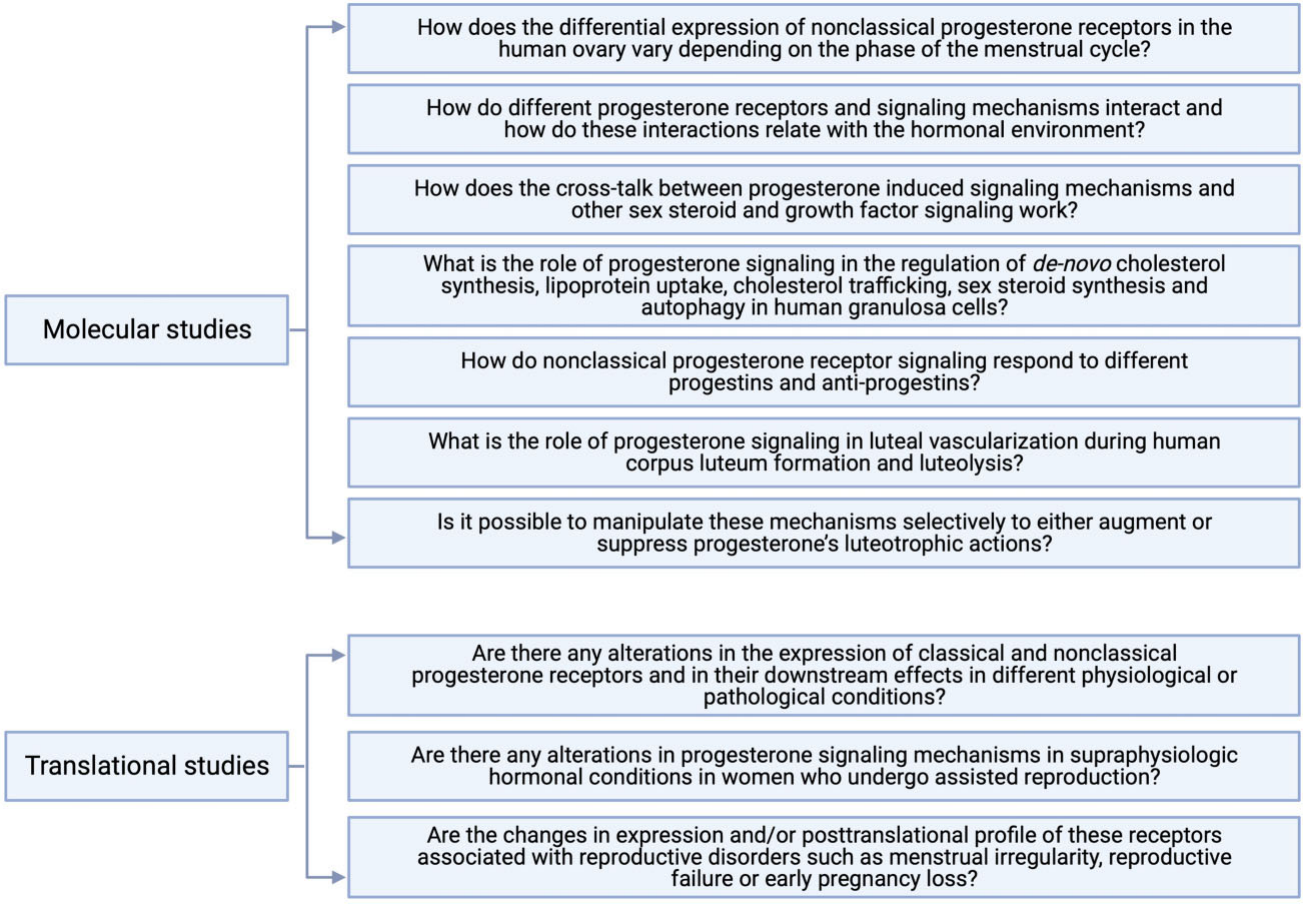
Major gaps in our understanding and future research priorities regarding progesterone signaling in the regulation of luteal steroidogenesis.

There is a pressing need for studies to demonstrate the molecular mechanisms of how progesterone manages to control the time-, site-, and context-dependent gene expression in target cells. There are major gaps in our knowledge regarding progesterone-induced signaling and progesterone’s luteotrophic activity. The overwhelming majority of published data is derived from animal models and they remain to be replicated in the human ovary. Figure 4 illustrates a list of research questions that necessitate further molecular and translational studies. However, the list is by no means exhaustive. A better understanding of the molecular mechanisms of progesterone-mediated functions is key to understanding luteal physiology, to illuminate the pathophysiology of luteal disorders, and to develop safe and effective strategies for their clinical management.

## Data Availability

No new data were generated or analyzed in support of this article.
